# Supplementary material for: Factors Associated with Impact of Event Scores Among Ontario Education Workers During the COVID-19 Pandemic
Source: Int J Environ Res Public Health. 2024 Oct 31;21(11):1448. doi: 10.3390/ijerph21111448 (PMC11593698; doi:10.3390/ijerph21111448)
Supplement: Supplementary file 1 [file ijerph-21-01448-s001.zip › ijerph-3198199-supplementary.pdf]

International Journal of
Environmental Research and Public Health

MDPI

Factors associated with Impact of Event scores among Ontario education workers during the COVID-19 pandemic

Supplementary Materials

Table S1. Impact of Event Scale item scores, Ontario education workers (September 2022-December 2023).

Sub-scale	Item	Median (IQR)
I	I thought about COVID-19 when I didn't mean to	3 (1, 3)
A	I avoided letting myself get upset when I thought about it or was reminded of it	3 (1, 5)
A	I tried to remove it from memory	1 (0, 3)
I	I had trouble falling asleep or staying asleep because of pictures or thoughts about it that came into my mind	0 (0, 1)
I	I had waves of strong feeling about it	0 (1, 3)
I	I had dreams about it	0 (0, 1)
A	I stayed away from reminders of it	0 (0, 3)
A	I felt as if it hadn't happened or it wasn't real	0 (0, 1)
A	I tried not to talk about it	0 (0, 3)
I	Pictures about it popped into my mind	1 (0, 3)
I	Other things kept making me think about it	1 (0, 3)
A	I was aware that I still had a lot of feelings about it, but I didn't deal with them	0 (0, 3)
A	I tried not to think about it	1 (0, 3)
I	Any reminder brought back feeling about it	1 (0, 3)
A	My feelings about it were kind of numb	1 (0, 3)

I: Intrusion subscale item; A: Avoidance subscale item; Item scoring: 0: not at all; 1: rarely; 3: sometimes; 5: often).

Table S2. Modified Poisson regression comparing Impact of Event subscale scores indicative of a subclinical/mild response versus moderate/severe response, Ontario education workers (September 2022-December 2023); Incidence rate ratio (95% CI).

Variable	Avoidance model, adjusted [1]	Intrusion model, adjusted [1]
Subjective health		
Poor/fair/good	Referent	Referent
Very good/excellent	**0.80 (0.69, 0.93)**	**0.74 (0.64, 0.85)**
Household size	NA	**1.09 (1.03, 1.15)**
Wears mask at work		
Never/rarely	Referent	Referent
Occasionally	1.07 (0.91, 1.26)	**1.29 (1.08, 1.55)**
Usually/always	0.78 (0.60, 1.01) *	**1.88 (1.55, 2.29)**
Physical distancing		
Never/rarely	Referent	Referent
Occasionally	**1.25 (1.04, 1.50)**	1.19 (0.98, 1.44)*
Usually/always	**1.43 (1.13, 1.80)**	**1.39 (1.10, 1.75)**
COVID-19, number of vaccines received	NA	**1.10 (1.04, 1.17)**
Hours worked	NA	**1.01 (1.00, 1.02)**
Highest level of student contact		
None /room >2 meters	NA	Referent
Room <2 meters		1.25 (0.95, 1.64)*
Physical contact		1.26 (0.95, 1.69)*

Bold: statistically significant ($p < 0.05$); * $p \leq 0.20$; NA: not applicable; 1: Adjusted for other variables in column and confounders: age and gender.
